# Soluble Tumor Necrosis Factor Receptor Mediates Cell Proliferation on Lipopolysaccharide-Stimulated Cultured Human Decidual Stromal Cells

**DOI:** 10.3390/ijms10052010

**Published:** 2009-05-04

**Authors:** Xue-Wen Yu, Xin-Wen Zhang, Xu Li

**Affiliations:** 1 Center of Maternal and Child Health, First Affiliated Hospital of Medical School in Xi’an Jiaotong University, Xi’an, 710061, P. R. China; E-Mails: yuxuewen@hotmail.com (X.-W.Y.); xianxinwen@163.com (X.-W.Z.); 2 Center of Molecular Biological Medicine, First Affiliated Hospital of Medical School in Xi’an Jiaotong University, Xi’an, 710061, P. R. China

**Keywords:** Decidual stromal cell (DSC), soluble tumor necrosis factor receptor 1 (sTNFR1), membrane tumor necrosis factor receptor 1 (mTNFR1), pregnancy, lipopolysaccharide (LPS)

## Abstract

The tumor necrosis factor-alpha (TNF-α) cytokine receptor system modulates apoptosis in many cell types, so we have investigated the role of sTNFR1 in bacterial lipopolysaccharide (LPS)-induced cell death in cultured human decidual stromal cells, hypothesizing that sTNFR1 might play a central role in this action. In this work we characterized *in vitro* decidual stromal cell viability with LPS treatment and LPS and sTNFR1 co-treatment. We found that LPS treatment induced decidual stromal cell death in a dose-dependent manner and that sTNFR1 blocked the effect of the LPS treatment. There was a significant proliferation among cells co-incubated with LPS at 10 μg/mL and sTNFR1 at 0.1 μg/mL compared with LPS and sTNFR1 at 0.01, 0.05, 0.2 and 0.5 μg/mL (p < 0.01). This study demonstrated that LPS led to decidual stromal cell death in *vitro* but sTNFR1 down-regulates the cell death due to LPS under the same conditions. Taken together, these results suggested that sTNFR1 could participate in a protective mechanism against endotoxin.

## 1. Introduction

The immune system is tightly regulated during pregnancy in order to avoid rejection of the semi-allogenic fetus, and this seems to contribute to the development of a normal pregnancy. Cytokines are believed to be important in maintaining pregnancy and in the initiation of labor in humans. Tumor necrosis factor-alpha (TNF-α) is a multifunctional Th1 cytokine with roles in regulating hormone synthesis, placental architecture, and embryonic development [1]. Increased placental TNF-α levels have been associated with pregnancy failure in mice [2], and elevated serum TNF-α levels are associated with the first-trimester threatened abortion-complicated pregnancies with adverse outcome in humans [3]. The administration of TNF-α increases abortion rates [4], and blockage of TNF-α has been identified as a potential therapy for the pregnancy complications of antiphospholipid syndrome in murine abortion models [5]. It is believed that TNF-α promotes apoptotic cell death in fetal membrane tissues [6] and prolonged or excessive production of TNF-α represents an important etiologic factor in inflammatory-based tissue injury [7].

During early pregnancy in mice, the implantation sites are highly sensitive to proinflammatory molecules such as lipopolysaccharide (LPS) [8]. LPS is a toxic component of the cell walls of Gram-negative bacteria, and has been associated with adverse developmental outcome, including embryonic resorption and intra-uterine fetal death in rodents [9]. Numerous studies show that LPS administration resulted in increase of TNF-α in maternal serum, amniotic fluid and placenta [10–12]. Indeed, in response to LPS, TNF-α is produced in large amounts earlier than any other cytokine. TNF-α has been associated with LPS-induced fetal loss and growth restriction in remaining viable fetuses [13].

TNF has two different receptors, a 55-kDa (TNFR1) and a 75-kDa one (TNFR2). Both types of TNFRs are shed from the cell surface and exist as soluble receptors. TNF and soluble TNFRs (sTNFRs) compose the system that relates with both Th1 and Th2 types of immune response because sTNFRs neutralize TNF. It has been found that TNF-activity can be inhibited *in vivo* and *in vitro* by administration of sTNFR (sTNFr-IgG), as demonstrated by successful inhibition of tissue damage in experimental autoimmune uveoretinitis [14]. Injection of sTNFRs neutralized TNF and abortion rate was reduced in mice [15]. These observations led us to hypothesize that the ability of LPS to induce embryonic resorption can be suppressed by sTNFR1 in humans. To test this hypothesis, the role of sTNFR1 in LPS-induced cell viability in cultured human DSCs was investigated. This anti-TNF treatment increased the decidual stromal cell (DSC) viability *in vitro*.

## 2. Results and Discussion

### 2.1. Suppression of viability /survival of DSCs by LPS in a dose-dependent manner

**Figure 1.**
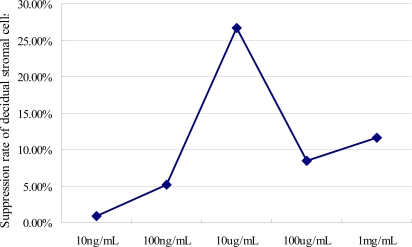
A dose-dependent effect of LPS on suppression of DSC viability by MTT test. The DSCs were cultured for 24 h in the presence of various concentrations of LPS. The value represents cell suppression rate of five independent experiments.

LPS in concentrations from 10 ng/mL to 1 mg/mL significantly suppressed viability of DSCs. Thus, the mean ± SEM spectrophotographic optical density for the cultured DSCs when exposed to various concentrations of LPS was 0.42 ± 0.03, 0.39 ± 0.01, 0.30 ± 0.02, 0.37 ± 0.01, 0.36 ± 0.02 at 10 ng/mL, 100 ng/mL, 10 μg/mL, 100 μg/mL, and 1 mg/mL of LPS, respectively. Figure 1 shows data on the rate of suppression of DSCs cultured in the presence of various concentrations of LPS for 24 h. The concentration of LPS that provided a most decreased viability of DSCs was 10 μg/mL. Thus, this concentration was selected for use in the final series of experiments.

### 2.2. Viability /Survival of DSCs by sTNFR1 in a dose-dependent manner

Co-incubated of DSCs with LPS and sTNFR1 for 24 h resulted in cell outgrowth. The mean ± SEM optical density for the cultured DSCs when treated with a combination of LPS at 10 μg/mL and sTNFR1 was 0.43 ± 0.01, 0.43 ± 0.01, 0.49 ± 0.02, 0.41 ± 0.15, 0.38 ± 0.15 at 0.01 μg/mL, 0.05 μg/mL, 0.1 μg/mL, 0.2 μg/mL, 0.5 μg/mL of sTNFR1, respectively.

**Figure 2.**
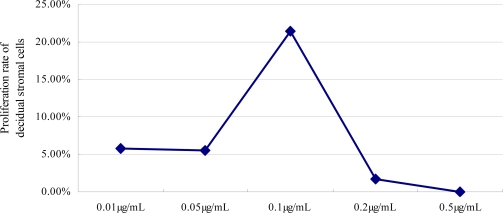
A dose-dependent effect of sTNFR1 on survival of DSCs by MTT test. The DSCs were cultured for 24 h in the presence of 10 ug/mL of LPS with various concentrations of sTNFR1 (0.01 μg/mL, 0.05 μg/mL, 0.1 μg/mL, 0.2 μg/mL, 0.5 μg/mL each). The value represents cell proliferation rate of five independent experiments.

sTNFR1 thus suppressed the decrease in DSC viability induced by LPS. The concentration of sTNFR1 that provided a statistically significant viability rate of DSCs was 0.1 μg/mL (p < 0.01) (Figure 2).

### 2.3. TNFR1 expression on cultured DSCs

**Figure 3.**
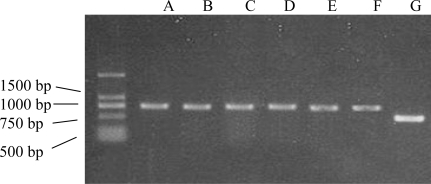
mRNA expression of TNFRI in cultural decidual stromal cells in response to lipopolysaccharide (LPS). An inverted image of a representative RT-PCR reaction is shown in the left six panels (747 bp). A, unstimulated cells; B, 1 mg/mL of LPS; C, 100 μg/mL of LPS; D, 10 μg/mL of LPS; E, 100 ng/mL of LPS; F, 10 ng/mL of LPS. The right panel (G) is GAPDH (500 bp).

To examine whether cell death is caused by LPS through regulation of TNFR1 expression, we performed RT-PCR and immunohistochemistry analysis to measure the expression of TNFR1 both at the protein and mRNA level. For the expression of TNFR1 at the mRNA level, RT-PCR was performed, using specific primers for TNFR1; primers for GAPDH were used as internal controls. Unstimulated DSCs expressed low levels of mTNFR1 mRNA (TNFR1 / GAPDH = 0.52 ± 0.045), and this was no further increase or decrease with exposure to various concentrations of LPS (Figure 3). For the expression of TNFR1 at the protein level, DSCs were stained with polyclonal antibody to TNFR1. A brown-yellow reaction product was observed in the cellular membrane and cytoplasm of DSCs. But, compared to unstimulated DSCs, no significant difference of mTNFR1 positive staining intensity was observed in DSCs following treatment with various concentrations of LPS. The sTNFR1 did not increase or decrease TNFR1 mRNA and mTNFR1 protein expression. These data suggested that the protective effect of sTNFR1 isn’t related to up-regulation or down-regulation of mTNFR1.

### 2.4. Discussion

Investigation of decidual cells *in vitro* provides a powerful approach to the study of the maternal environment and its role in the establishment and maintenance of pregnancy. DSCs are one of the principle cell types found in the placenta. To study how bacteria may affect DSCs, we cultured DSCs treated for 24 h with LPS and examined DSC proliferation suppression. Results with the MTT assay showed stimulation more than suppression in DSC proliferation, demonstrating that LPS has a potent *in vitro* effect on inhibiting cell proliferation in this important DSC type.

As the TNF-α cytokine receptor system has been shown to modulate apoptosis in many cell types [16–18], we cultured DSCs from normal first-trimester pregnant females and determined mTNFR1 response to a bacterial product. The results described in this study show that TNFR1 expression at the mRNA and protein level is present in unstimulated DSCs, in accordance with observations reported by Arntzen and Menon [19,20]. But the up-regulation of mTNFR1 does not take place for cells to respond to LPS stimulation in this study. It is not clear that there is biologic plausibility to a bacterial product response of mTNFR1 in human decidua.

In this series of experiments, we find that sTNFR1 modulates the proliferation of cultured human DSCs in response to the potent bacterial product LPS. The sTNFR1 increases the DSC proliferation from the first-trimester pregnancy undergoing LPS stimulation, and suggests that sTNFR1 could participate in a protective mechanism against endotoxin. We also find that 0.1 μg/mL concentration of sTNFR1 provided the greatest proliferation rate of DSCs. There is no increased viability of DSCs with an increasing concentration of sTNFR1, which may explain the inconsistent *in vivo* serum sTNFR1 level results of women with early spontaneous miscarriages [21,22].

It has been found that explants from both amnion and choriodecidua produce TNF upon LPS stimulation, and LPS increased production of TNF from decidual cells with a subsequent release prostaglandins [19,23]. Reduction of the biological activities of TNF can be accomplished by several different, but highly specific strategies, which involve soluble receptors, receptor antagonists and inhibitors of proteases that convert inactive precursors to active, mature molecules. The sTNFR1 represents the extracellular domains of the TNFR1. After shedding by proteinase TNF-α converting enzyme, the sTNFR1 circulates in many body fluids and retains its ability to bind circulating TNF-α. Depending on their relative concentrations, sTNFRs have been suggested to either block TNF-α binding to mTNFRs and thus the subsequent biological responses [24] or to enhance TNF-α action by stabilization of the homotrimeric cytokine [25]. Elevated sTNFR1 level has been found in serum in association with inflammatory and infectious diseases, suggesting a direct or indirect implication of the TNF system in the pathophysiology of these diseases [26,27]. Since decidua is most to be involved at an early point in the host immune response to ascending microbial invasion of the upper genital tract, understanding and modulating decidual immune response is of critical importance. For this reason, our finding of a promotion of DSC death in response to bacterial products and a reduction of DSC death after incubation with sTNFR1 suggests that sTNFR1 may be a useful immunomodulatory agent in the therapies for infection-related, early spontaneous miscarriage. Future investigations exploring the immunomodulatory role of sTNFR1 in an animal model of infection-induced spontaneous miscarriage are warranted.

## 3. Materials and Methods

### 3.1. Cell cultures

Decidual specimens from elective terminations between 7 and 10 weeks of gestation were obtained under Department of Science and Research approval at the First Hospital of Xi’an Jiaotong University Medical School, P.R China. Tissues were rinsed with 0.9% saline to remove blood cells and mucus. Then, the tissues were minced and digested with 0.25% trypsin, as well as 20 mg/L DNase in RPMI 1640 medium containing 100 U/mL penicillin and 100 μg/mL streptomycin (Hyclone Co, America) in a 37 °C shaking water bath for 2 h. After washing with sterile phosphate-buffered saline (PBS), the digestate was washed three times and subjected to consecutive filtration through 100 μm and 200 μm Millipore filters, respectively. The filtrate was centrifuged at 2,000 g for 3 min. The pellet of mixed cells was washed twice and resuspended in RPMI 1,640 containing 100 U/mL penicillin, 100 μg/mL streptomycin and 10% fetal calf serum and seeded on polystyrene tissue culture dishes, grown to confluence in a standard 95% air: 5% CO_2_ incubator at 37 °C. After five passages, by standard methods of trypsin treatment, the cells were > 95% pure as determined by immunohistochemical staining with antibodies to vimentin. These cells were used for the experiments.

### 3.2. Incubation experiments

Using human DSC cultures, we placed 0.125 × 10^6^ cells in each well of a 12-well plate and let them adhere overnight. The cells were treated for 24 h with LPS at 1 mg/mL, 100 μg/mL, 10 μg/mL, 100 ng/mL, and 10 ng/mL and dose–response experiments were performed. A series of 24 h incubations were performed under both LPS at 10 μg/mL and sTNFR1 at 0.01 μg/mL, 0.05 μg/mL, 0.1 μg/mL, 0.2 μg/mL, 0.5 μg/mL each. Cellular viability was confirmed with each dose condition using a MTT [3-(4,5-dimethylthiazole-2-yl)-2,5-diphenyl tetrazolioum bromide] assay according to the manufacturer’s instructions.

### 3.3. TNFR1 mRNA of DSCs by reverse transcription-PCR (RT-PCR)

The extraction of total cellular RNA was based on the single-step method. Cells were lysed by the addition of 1.0 mL of TRI-reagent solution (Invitrogen, USA) for every 1 × 10^6^ cells and solubilized by passing the lysate through a l mL pipette. RNA was extracted and precipitated according to the manufacturer’s instructions. The final pellet was resuspended in 50 μL DEPC. First strand DNA was synthesized at 42 °C for 60 min using 1 μg of total RNA, 1 μL of AMV reverse transcriptase (Fermentas, USA), 1 μL of oligo(dT) primer, dNTP (0.5 mM each of dGTP, dATP, dTTP, and dCTP) and 4 μL of MgCl2 in RT buffer. The DNA was then amplified using 1 μL of Taq DNA polymerase (Tiangen, German), 1 μL of sequence-specific primers against human TNFR1 (sense, 5’-ATCTCTATGCCCGAGTCTCAACC-3’; antisense, 5’-CTCAATCTGGGGTAGGCACAAC-3’ for 727 bp products), in PCR buffer (20 mM Tris-HCl, pH 8.4, 50 mM KC1 and 2 mM MgCl2) for 28 cycles by PCR Thermal Cycle (FORMA Co. USA). The amplification profile involved denaturation at 94 °C for 30 sec, primer annealing at 57 °C for 30 sec, and extension at 72 °C for 30 sec. Primers for GAPDH (sense, 5’-AGCCAAAAGGGTCATCATCT-3’; antisense, 5’-CCACCTGGTGCTCAGTGT AG-3’ for 500 bp) were added in the same tube, and the amount of GAPDH cDNA generated was used as an internal control. A 20 μL sample of a 50 μL PCR mixture was electrophoresed on a 1% agarose gel, stained in ethidium bromide, and photographed by the image analysis system. Levels of TNFR1 mRNA were calculated in arbitrary units as the proportion of TNFR1 PCR product intensity to GADPH PCR product intensity from the same RNA sample.

### 3.4. Membrane TNFR1 (mTNFR1) protein of DSCs by Immunohistochemistry

DSCs growing on 2-well chamber slides were fixed for 10 min in 4% paraformaldehyde (PFA)/0.1 PBS (pH 7.2 – 7.6), washed twice with PBS and permeabilized for 8 min with 0.1% Triton X-100/PBS. The fixed cells were incubated with a rabbit polyclonal anti-human TNFR1 antibody diluted at 1 : 25 (Wuhan Boster Biotechnology Co., China) and a mouse monoclonal anti-human vimentin antibody diluted at 1:500 (DAKO, Denmark) for 2 hours at 37 °C, followed by incubation with biotinylated goat anti-rabbit IgG and goat anti mouse IgG for 30 min. For the negative control, the primary antibody was omitted. The cells were then incubated in DAB reagent and counterstained with hematoxylin and coverslipped using Protexx mounting media (DAB-Stock Stain box; Zymed Laboratories, USA). A positive reaction for mTNFR1 protein was defined as a brown-yellow granulation in the cellular membrane and cytoplasm. Negative cells had clear cell structure without brown granulation in their cellular membrane and cytoplasm.

### 3.5. Statistical analysis

The results of cellular viable assay and mTNFR1 expression from three experiments were presented as mean ± SEM. A two-sample assuming equal variances Student t-test was applied to test the statistical significance where p < 0.05.

## 4. Conclusions

In this study we have demonstrated that LPS led to decidual stromal cell death *in vitro*, but sTNFR1 increased the DSC proliferation from the first trimester of pregnancy following LPS stimulation. Taken together, these results suggested that sTNFR1 could participate in a protective mechanism against LPS endotoxin.

## References

[1] Toder V, Fein A, Carp H, Torchinsky A (2003). TNF-α in pregnancy loss and embryo maldevelopment: a mediator of detrimental stimuli or a protector of the fetoplacental unit?. J. Assist. Reprod. Genet.

[2] Fest S, Zenclussen AC, Joachim R, Hagen E, Demuth HU, Hoffmann T (2006). Stress and substance P but not the substance P-metabolite SP5-11 trigger murine abortion by augmenting TNF-alpha levels at the feto-maternal interface. Scand. J. Immunol.

[3] Vitoratos N, Papadias C, Economou E, Makrakis E, Panoulis C, Creatsas G (2006). Elevated circulating IL-1β and TNF-alpha, and unaltered IL-6 in first-trimester pregnancies complicated by threatened abortion with an adverse outcome. Mediators Inflamm.

[4] Clark DA, Manuel J, Lee L, Chaouat G, Gorczynski RM, Levy GA (2004). Ecology of danger-dependent cytokine-boosted spontaneous abortion in the CBA x DBA/2 mouse model. I. Synergistic effect of LPS and (TNF-alpha + IFN-gamma) on pregnancy loss. Am. J. Reprod. Immunol.

[5] Berman J, Girardi G, Salmon JE (2005). TNF-α is a critical effector and a target for therapy in antiphospholipid antibody-induced pregnancy loss. J. Immunol.

[6] Fortunato SJ, Menon R (2003). IL-1 β is a better inducer of apoptosis in human fetal membranes than IL-6. Placenta.

[7] Graves DT (1999). The potential role of chemokines and inflammatory cytokines in periodontal disease progression. Clin. Infect. Dis.

[8] Ogando DG, Paz D, Cella M, Franchi AM (2003). The fundamental role of increased production of nitric oxide in lipopolysaccharide-induced embryonic resorption in mice. Reproduction.

[9] Buhimschi IA, Buhimschi CS, Weiner CP (2003). Protective effect of N-acetylcysteine against fetal fetal death and preterm labor induced by maternal inflammation. Am. J. Obstet. Gynecol.

[10] Bell MJ, Hallenbeck JM, Gallo V (2004). Determining the fetal inflammatory response in an experimental model of intrauterine inflammation in rats. Pediatr. Res.

[11] Gayle DA, Beloosesky R, Desa M, Amidi F, Nunez SE, Ross MG (2004). Maternal LPS induces cytokines in the amniotic fluid and corticotropin releasing hormone in the fetal rat brain. Am. J. Physiol. Regul. Integr. Comp. Physiol.

[12] Holcberg G, Amash A, Sapir O, Sheiner E, Levy S, Huleih M (2007). Perfusion with lipopolysaccharide differently affects the secretion of tumor necrosis factor-and interleukin-6 by term and preterm human placenta. J. Reprod. Immunol.

[13] Robertson SA, Care AS, Skinner RJ (2007). Interleukin 10 regulates inflammatory cytokine synthesis to protect against lipopolysaccharide-induced abortion and fetal growth restriction in mice. Biol. Reprod.

[14] Robertson M, Liversidge J, Forrester JV, Dick AD (2003). Neutralizing tumor necrosis factor activity suppresses activation of infiltrating macrophages in experimental autoimmune uveoretinitis. Invest. Ophthalmol. Vis. Sci.

[15] Arck PC, Troutt AB, Clark DA (1997). Soluble receptors neutralizing TNF-alpha and IL-1 blockstress-triggered murine abortion. Am. J. Reprod. Immunol.

[16] Alexander JJ, Jacob A, Cunningham P, Hensley L, Quigg RJ (2008). TNF is a key mediator of septic encephalopathy acting through its receptor, TNF receptor-1. Neurochem. Inter.

[17] Aggarwal S, Gollapudi S, Yel L, Gupta AS, Gupta S (2000). TNF-a-induced apoptosis in neonatal lymphocytes: TNFRp55 expression and downstream pathways of apoptosis. Genes Immun.

[18] Takada Y, Sung B, Sethi G, Chaturvedi MM, Aggarwal BB (2007). Evidence that genetic deletion of the TNF receptor p60 or p80 inhibits Fas mediated apoptosis in macrophages. Biochem. Pharmaco.

[19] Arntzen KJ, Egeberg K, Rahimipoor S, Vatten L, Austgulen R (1999). LPS mediated production of IL-1, PGE2 and PGF2a from term decidua involves tumour necrosis factor and tumour necrosis factor receptor p55. J. Reprod. Immunol.

[20] Menon R, Thorsen P, Vogel I, Jacobsson B, Williams SM, Fortunato SJ (2007). Increased bioavailability of TNF-alpha in African Americans during *in vitro* infection: predisposing evidence for immune imbalance. Placenta.

[21] Chernyshov VP, Vodyanik MA, Pisareva SP (2005). Lack of soluble TNF-receptors in women with recurrent spontaneous abortion and possibility for its correction. Am. J. Reprod. Immunol.

[22] Yu XW, Yan CF, Jin H, Li X (2005). Tumor necrosis factor receptor 1 expression and early spontaneous abortion. Int. J. Gynecol. Obstet.

[23] Leroy MJ, Dallot E, Czerkiewicz I, Schmitz T, Breuiller-Fouché M (2007). Inflammation of choriodecidua induces tumor necrosis factor alpha-mediated apoptosis of human myometrial cells. Biol. Reprod.

[24] VanZee KJ, Kohno T, Fischer E, Rock CS, Moldawer LL, Lowry SF (1992). Tumor necrosis factor soluble receptors circulate during experimental and clinical inflammation and can protect against excessive tumor necrosis factor alpha *in vitro* and *in vivo*. Proc. Natl. Acad. Sci. USA.

[25] Kohno T, Brewer MT, Baker SL, Schwartz PE, King MW, Hale KK, Squires CH, Thompson RC, Vannice JL (1990). A second tumor necrosis factor receptor gene product can shed a naturally occurring tumor necrosis factor inhibitor. Proc. Natl. Acad. Sci. USA.

[26] Athanassakis I, Aifantis I, Ranella A, Giouremou K, Vassiliadis S (1999). Inhibition of nitric oxide production rescues LPS-induced fetal abortion in mice. Biol. Chem.

[27] Bilello JA, Stellrecht K, Drusano GL, Stein DS (1996). Soluble tumor necrosis factor-alpha receptor type II (sTNF alpha RII) correlates with human immunodeficiency virus (HIV) RNA copy number in HIV-infected patients. J. Infect. Dis.

